# Neurochagas: Experimental and human literature of an uncommon entity (Review)

**DOI:** 10.3892/mi.2026.320

**Published:** 2026-05-11

**Authors:** Jamir Pitton Rissardo, Hossam Tharwat Ali, Vishnu Vardhan Byroju, Ana Leticia Fornari Caprara

**Affiliations:** 1 Department of Neurology, Cooper University Hospital, Camden, NJ 08103, USA; 2 Neuropsychiatry Department, Qena Faculty of Medicine, Qena University, Qena 83621, Egypt; 3 Department of Medicine, Federal University of Santa Maria, Santa Maria, RS 97105110, Brazil

**Keywords:** Chagas disease, *Trypanosoma cruzi*, neurochagas, central nervous system, encephalitis

## Abstract

Neurochagas, the neurological manifestation of Chagas disease, presents a challenge to global health, affecting millions of individuals worldwide. Despite significant progress being made in the understanding of the pathogenesis and clinical presentation of neurochagas, numerous gaps persist in the literature, hindering effective diagnosis and management. The present review discusses the relevant experimental and human literature regarding neurochagas and its epidemiology, pathophysiology, clinical manifestations, diagnostic approaches, treatment strategies and future directions for research. Neurochagas includes a broad spectrum of neurological manifestations, including meningoencephalitis, stroke-like syndromes, cognitive impairment, movement disorders, peripheral neuropathy and autonomic dysfunction, resulting from *Trypanosoma cruzi* invasion and neuroinflammatory responses. Diagnosing this neurozoonotic disease relies on a multidimensional approach, incorporating clinical evaluation, serological testing, neuroimaging studies, cerebrospinal fluid analysis and ancillary tests. Treatment strategies for neurochagas focus on antiparasitic therapy, symptomatic management, immunomodulation and supportive care. However, available clinical evidence and evidence of proper nervous system penetration is limited, particularly for advanced neurological complications. Future studies are required in order to advance the understanding of the disease mechanisms, identifying biomarkers for early diagnosis and treatment response assessment, developing novel therapeutic interventions, implementing precision medicine approaches, and strengthening global health initiatives to reduce the burden of neurochagas.

## 1. Introduction

**Figure 1:**
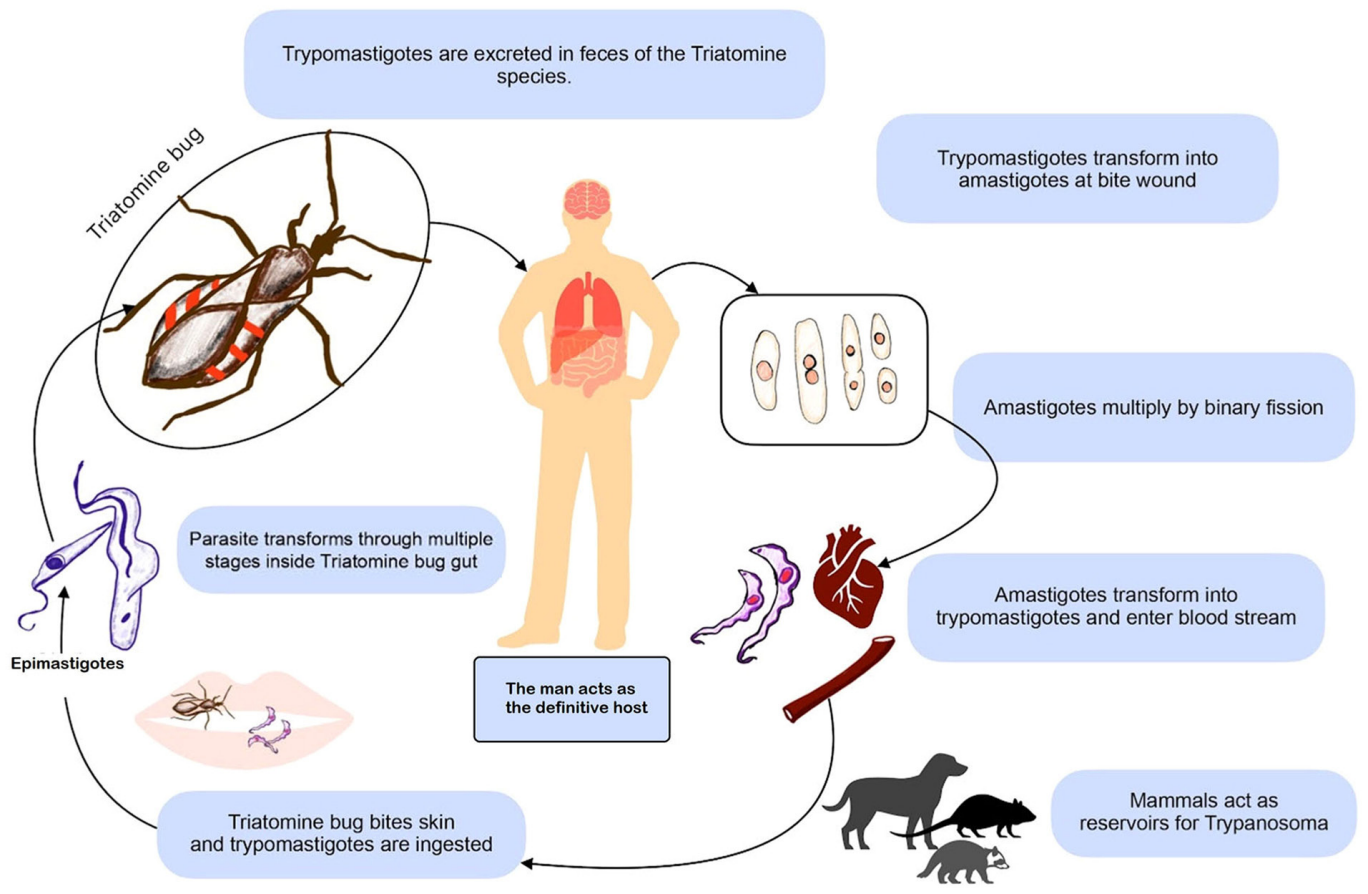
Life cycle of *Trypanosoma cruzi*. Kissing bug bites infect humans and trypomastigotes, which undergo reproductive cycles in the bug’s gut, are ingested. This bug excretes these parasites into the wound site during a meal. Replication inside the human host occurs by binary fission, and trypomastigotes circulate across the bloodstream. The figure is author-generated.

Chagas disease is caused by the protozoan parasite, *Trypanosoma cruzi* (*T. cruzi*), classified into seven genotypes known as discrete typing units (1). Traditionally acknowledged for its cardiac and gastrointestinal implications, Chagas disease harbors a lesser-known, yet equally formidable involvement of the neurological system. The lifecycle of the parasite is illustrated in Fig. 1. The geographic footprint of Chagas disease extends across Latin America, where it remains endemic, and contributes to other names for this condition, such as ‘American trypanosomiasis’. However, globalization and human migration have facilitated the dissemination to non-endemic regions, amplifying its global significance. Neurochagas is often underrecognized due to its subtle and heterogeneous presentation (2,3). The ability of the parasite to breach the blood-brain barrier (BBB) and invade the neurological system underscores the intricate interplay between host and pathogen. Furthermore, neurochagas may lead to immune-mediated processes, culminating in neuroinflammation and neurodegeneration. Understanding these intricate pathways is crucial for developing targeted therapeutic interventions (4,5).

From meningoencephalitis to movement disorders, cognitive impairment and psychiatric symptoms, neurochagas casts a wide net, involving both the central and peripheral nervous systems. These clinical phenotypes, often overlapping with other neurological conditions, are complex to diagnose, and this highlights the importance of a high clinical suspicion in the differential diagnosis (6,7). In addition to the combination of non-specific clinical presentation, there are limited diagnostic tools. Leveraging a multidimensional approach encompassing clinical evaluation, laboratory investigations, neuroimaging studies and cerebrospinal fluid analysis is imperative for an accurate diagnosis and timely intervention (7,8).

Therapeutic strategies for neurochagas remain limited, with antiparasitic agents serving as the mainstay of treatment. However, challenges persist in accessing and tolerating these medications, particularly in the context of neurological complications, as some of the available therapies can worsen neurological symptoms. In addition, supportive management strives to ameliorate neurological deficits and enhance the quality of life of affected individuals; however, gaps persist in addressing the underlying pathophysiological mechanisms driving neurochagas (4,9). Overall, drawbacks and a lack of sufficient evidence exist with regard to diagnosis, pathophysiology and the management of neurochagas. Therefore, the present review aimed to discusses the available literature regarding the neurological manifestations of Chagas disease and its epidemiological features, pathophysiology and management.

## 2. Literature search

Aiming to comprehensively and narratively review the literature, a search of the PubMed/Medline database for relevant articles was performed using key words such as ‘Neurochagas’, ‘Chagas disease’, ‘Trypanosoma’ and ‘American Trypanosomiasis’ to the date of September, 2025, with the filter of English language. No other specific criteria for inclusion or exclusion were applied.

## 3. Epidemiology of neurochagas

### Geographical distribution

The geographic distribution of Chagas disease, caused by the protozoan parasite *T. cruzi*, is predominantly concentrated in Latin America, where it remains endemic. However, globalization, migration and changing ecological factors have facilitated its spread to non-endemic regions, making it a global health concern. The highest prevalence rates are observed in rural areas where poor housing conditions and inadequate access to healthcare services create conducive environments for transmission. Triatomine bugs, the primary vectors of *T. cruzi*, thrive in these settings, nesting in the cracks and crevices of substandard housing and feeding on human blood during the night (6,10). Within Latin America, the distribution of Chagas disease exhibits considerable heterogeneity, with some regions experiencing higher prevalence rates than others. The ‘Gran Chaco region’, spanning parts of Argentina, Bolivia and Paraguay, is one of the most heavily affected areas, with prevalence rates >20% in some communities. Other endemic regions include the Amazon basin, the Andean highlands and parts of Central America, where environmental conditions favor the proliferation of vector populations (11,12).

Beyond Latin America, Chagas disease has gained recognition as an emerging health threat in non-endemic regions, including North America, Europe and the Western Pacific. Migration plays a pivotal role in the global dissemination of Chagas disease, as infected individuals carry the parasite to new geographical areas. In the USA, Chagas disease is increasingly recognized, particularly among immigrants from endemic countries. Cases have been reported in states with large immigrant populations, such as Texas, California and Florida (13,14). The Western Pacific region, encompassing countries, such as Australia and Japan, has also reported sporadic cases of Chagas disease, often linked to travel or migration from endemic areas. While transmission through local vector populations is rare in these regions, importing infected triatomine bugs or infected blood products poses a potential risk. Thus, the geographical distribution of Chagas disease spans across Latin America, where it remains endemic, and extends to non-endemic regions through migration and globalization (4,6).

Europe has also witnessed a rise in cases of Chagas disease, primarily attributed to migration from Latin America. Countries such as Spain, Italy and Switzerland have reported autochthonous cases, indicating local transmission through infected triatomine bugs or congenital transmission from infected mothers. Moreover, blood transfusion and organ transplantation have emerged as potential routes of Chagas disease transmission in non-endemic regions, underscoring the importance of screening blood donors and implementing stringent donor selection criteria (15,16). Therefore, the routes of *T. cruzi* infection are vector-borne transmission, blood transfusion, congenital or transplacental transmission from infected mothers to infants, organ transplants, the ingestion of foods contaminated with *T. cruzi*, and accidental infection in laboratories (4,6,10).

### Prevalence and incidence

The prevalence and incidence of neurochagas present complex challenges in estimation due to underreporting, misdiagnosis and varying degrees of disease severity. However, several studies have provided insight into the burden of neurochagas, particularly in endemic regions of Latin America (4,6,14,15). Estimates of the prevalence of neurochagas vary widely across different studies and regions, ranging from <1 to >30% among individuals with chronic Chagas disease, according to different reviews and cohorts, which reflects the vast difference between socioeconomic backgrounds in South America. A summary of the variable prevalence and incidence of Chagas disease according to geographical distribution is presented in Table I (4-6,8,14,15). Neurological complications may manifest years or even decades following the initial infection, contributing to the challenges of accurately estimating prevalence rates. Additionally, the diversity of neurological manifestations further complicates efforts to capture the entire burden of neurochagas. Incidence data for neurochagas are limited, primarily due to the need for longitudinal studies tracking new cases of neurological involvement over time. However, it is generally considered that the incidence of neurochagas is lower than the prevalence, reflecting the chronic and progressive nature of neurological complications in Chagas disease (4,6).

Several factors influence the prevalence and incidence of neurochagas, including the geographic distribution of *T. cruzi*, the virulence of parasite strains, host immune responses, and access to healthcare services for early diagnosis and treatment. Inadequate vector control measures and socio-economic disparities exacerbate the burden of Chagas disease, increasing the risk of neurological complications among affected populations (7,17).

### Risk factors for neurochagas

Identifying risk factors for neurochagas is crucial for understanding disease progression, guiding clinical management and implementing targeted preventive measures. While the exact mechanisms underlying neurochagas remain incompletely understood, several factors have been implicated in increasing the likelihood of neurological complications in Chagas disease. Prolonged exposure to *T. cruzi* is one of the most critical risk factors for developing neurological manifestations with Chagas disease. Chronic infection, often lasting decades, increases the likelihood of parasite invasion into neural tissues and subsequent neuroinflammatory responses. Chronic peripheral inflammation is considered to lead to decreased protection of the central nervous system (CNS), facilitating the dissemination of the parasite to central neural tissues (4,5,18).

Variability in the virulence and genetic diversity of *T. cruzi* strains may influence the risk of chronic infection and dissemination to the CNS. Some parasite genotypes have been associated with increased neurotropism and an enhanced ability to invade neural tissues. Furthermore, the host immune responses may play a critical role in determining the clinical course of Chagas disease, including the development of neurological manifestations (19-21). Dysregulated immune responses, characterized by the excessive inflammation or impaired immune surveillance, may exacerbate neuronal damage and contribute to the progression of neurochagas (21-23).

The age at which individuals acquire Chagas disease may influence their risk of developing neurological complications. Early childhood infections are often associated with milder disease manifestations, whereas infections acquired during adulthood may lead to more severe and symptomatic neurochagas. Moreover, underlying medical conditions, such as immunosuppression, diabetes mellitus and cardiovascular disease, can exacerbate the risk of developing neurological complications due to impairment in the immune function and vascular integrity, facilitating the dissemination of *T. cruzi* to the CNS (2,5,8,9,14,18).

Genetic factors may predispose some individuals to an increased risk of neurochagas. Polymorphisms in genes involved in immune regulation, neuronal function and parasite recognition have been implicated in modulating susceptibility to Chagas disease and its severe neurological manifestations. In addition, socioeconomic disparities, including poverty, inadequate housing and limited access to healthcare services, contribute to the burden of Chagas disease and increase the risk of developing neurological complications. Poor living conditions favor the proliferation of vector populations and enhance the likelihood of parasite transmission, particularly in endemic regions (2,4,5).

## 4. Pathophysiology of neurochagas

### Mechanisms of neural invasion

The mechanisms underlying the neural invasion of *T. cruzi* involve a complex interplay between the parasite, host immune responses and neural tissues. While the exact pathways of neural invasion remain incompletely understood, several mechanisms have been proposed. *T. cruzi* may exploit various strategies to disrupt the integrity of the BBB, including releasing proteases and inflammatory mediators, leading to increased permeability and facilitating its entry into neural tissues. *T. cruzi* exhibits neurotropism by recognizing specific surface receptors or exploiting chemotactic signals in the nervous system environment. Once inside neural tissues, *T. cruzi* can evade host immune surveillance and establish chronic infections, contributing to neurological complications (22,24,25).

**Figure 2:**
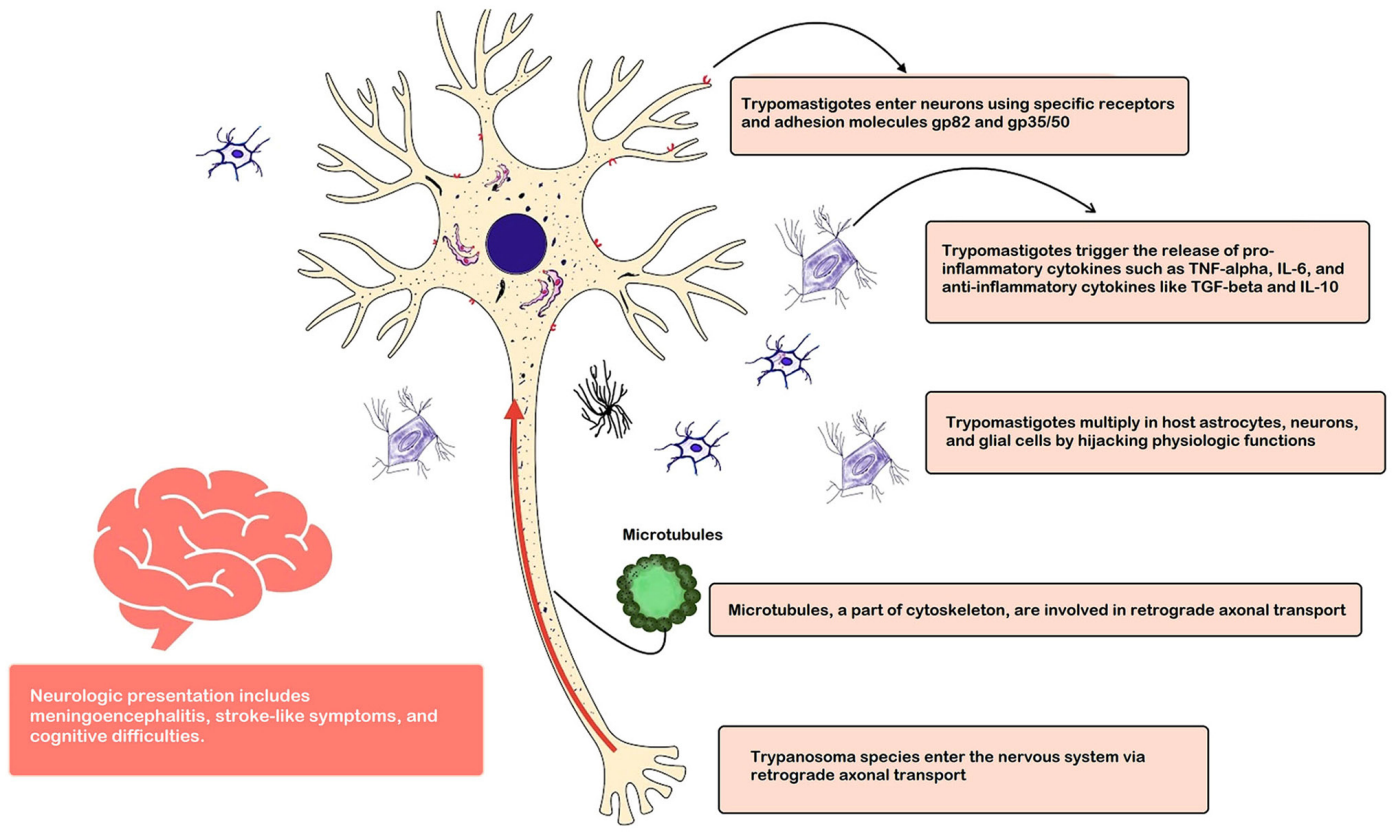
Neural invasion of *T. cruzi* and pathophysiology of neurochagas. As hypothesized from experimental models, *T. cruzi* species gain access to the central nervous system via retrograde axonal transport using microtubules, as well as adhesion molecules on the neuronal surface. While hijacking physiological function, *T. cruzi* enhances the release of inflammatory cytokines, which further contribute to the neuronal damage process. The figure is author-generated. *T. cruzi, Trypanosoma cruzi.*

As hypothesized from experimental models, *T. cruzi* may utilize retrograde axonal transport as a mechanism for neural invasion. Following entry into peripheral nerve endings or muscle cells, the parasite can hijack the transport machinery of the host cell, including microtubules and molecular motors, to travel along axons toward the CNS. This retrograde transport allows *T. cruzi* to bypass local immune defenses and gain access to neuronal cell bodies within the spinal cord or brain (26,27). In addition, *T. cruzi* can directly invade neurons through interactions with surface receptors and host cell signaling pathways. The parasite may utilize adhesion molecules, such as gp82 and gp35/50, to adhere to and penetrate neural cells, triggering intracellular signaling cascades that facilitate its internalization. Once inside neurons, *T. cruzi* can replicate and spread to neighboring cells, leading to neuronal damage and inflammation (28,29). The methods of the neural invasion of *T. cruzi* are illustrated in Fig. 2. The early invasion stage of peripheral nerves, as well as the inflammatory process, can give rise to peripheral neuropathy syndromes.

Another possible explanation for neuronal damage is the ‘Trojan horse’ strategy, in which *T. cruzi* enters the CNS by infecting circulating immune cells, such as monocytes and macrophages. These infected immune cells carry the parasite, allowing it to evade immune surveillance and disseminate to neural tissues upon extravasation from the bloodstream (30,31). Once inside the CNS, *T. cruzi* can infect resident glial cells and neurons, perpetuating chronic inflammation and tissue damage. Rodent models have raveled CNS abnormalities and parasite presence in the brain following *T. cruzi* infection, despite peripheral entry via the skin. The presence of the parasite in different brain regions has been demonstrated in animal models, as presented in Table II (10,17,19,20,32-36).

### Immune response and neuroinflammation

The immune response and neuroinflammation play pivotal roles in the pathogenesis of neurochagas, the neurological manifestations of Chagas disease. Upon the invasion of neural tissues by *T. cruzi*, the parasite responsible for Chagas disease, a cascade of immune-mediated processes is initiated, leading to neuroinflammation and neuronal damage. Molecular interactions occur between *T. cruzi* and host molecules in the CNS, as presented in Table III (21,23,25,32,37-39).

The innate immune system is the first line of defense against *T. cruzi* invasion in the CNS. Innate immune cells, such as microglia, astrocytes and infiltrating macrophages, recognize parasite-derived molecules, known as pathogen-associated molecular patterns, through pattern recognition receptors. The activation of these receptors triggers the production of pro-inflammatory cytokines, chemokines and reactive oxygen species, contributing to neuroinflammation and tissue damage (17,32,33). The adaptive immune response is critical in controlling *T. cruzi* infection and modulating neuroinflammatory processes in neurochagas. T-lymphocytes, including CD4^+^ T-helper cells and CD8^+^ cytotoxic T-cells, infiltrate neural tissues in response to parasite antigens and contribute to the clearance of infected cells. However, dysregulated T-cell responses, characterized by excessive inflammation or immune evasion, can exacerbate neuronal damage and contribute to the progression of neurochagas (17,32,33).

The dysregulated production of pro-inflammatory cytokines, such as tumor necrosis factor-alpha (TNF-α), interleukin (IL)-1β and IL-6, is a hallmark of neuroinflammation in neurochagas. These cytokines are secreted by activated immune cells and glial cells in response to *T. cruzi* infection and contribute to neuronal dysfunction, BBB disruption and tissue damage. Conversely, anti-inflammatory cytokines, such as IL-10 and transforming growth factor-β (TGF-β), may serve as regulatory mechanisms to dampen excessive inflammation and limit tissue injury (21,25,38,39).

Glial cells, including microglia and astrocytes, play crucial roles in neuroinflammation and neuroprotection in neurochagas. Upon activation by parasite-derived factors or inflammatory mediators, glial cells undergo morphological and functional changes, producing pro-inflammatory cytokines, chemokines and neurotoxic molecules. Additionally, activated glial cells can phagocytose *T. cruzi* parasites and infected cells, contributing to parasite clearance and tissue repair. Furthermore, disrupting the BBB allows immune cells, cytokines and neurotoxic molecules to infiltrate neural tissues, exacerbating neuroinflammation and neuronal damage (29,30,37,39).

### Impact on neuronal function and structure

*T. cruzi* can invade neural tissues, leading to a cascade of pathophysiological processes that disrupt neuronal homeostasis and integrity. *T. cruzi* infection can directly impair neuronal function by disrupting ion homeostasis, neurotransmitter release and synaptic transmission. The parasite invasion of neurons may lead to alterations in neuronal excitability, synaptic plasticity and neuronal network activity, contributing to neurological deficits (4,9,14).

Neurochagas is characterized by chronic neuroinflammation, the infiltration of immune cells, the activation of glial cells, and the production of pro-inflammatory cytokines and chemokines. Neuroinflammatory processes contribute to neuronal dysfunction and damage by releasing neurotoxic molecules, oxidative stress and excitotoxicity, leading to synaptic loss, dendritic remodeling and neuronal death. *T. cruzi* infection can cause axonal degeneration and demyelination, disrupting neuronal connectivity and impairing signal transmission. Axonal pathology may result from direct parasite invasion of axons, neuroinflammatory responses, or immune-mediated mechanisms (1,4,17,27,28).

Neuronal injury and apoptosis occur in neurochagas due to various pathogenic mechanisms, including excitotoxicity, mitochondrial dysfunction and oxidative stress. *T. cruzi* infection may induce neuronal apoptosis by activating intrinsic and extrinsic apoptotic pathways, leading to neuronal loss and gliosis. The extent of cellular and cerebrovascular affection determines the localization and nature of pathology, elucidating the clinical syndrome of the CNS. In addition, chronic neuroinflammation and neuronal injury in neurochagas can culminate in progressive neurodegeneration, characterized by the gradual loss of neurons and synaptic connections. Neurodegenerative changes may manifest as cortical atrophy, white matter lesions, and subcortical degeneration, contributing to cognitive decline, motor impairment and functional disability in affected individuals. Moreover, the disruption of the BBB in neurochagas facilitates the infiltration of immune cells, cytokines and neurotoxic molecules into neural tissues, exacerbating neuronal dysfunction and damage. BBB dysfunction may further compromise neuronal integrity and exacerbate neuroinflammatory responses, perpetuating a cycle of neuronal injury and dysfunction (4-7,9,14,35).

## 5. Clinical presentation

Chagas disease can be divided into acute and chronic phases. The acute phase usually occurs within the first 8 weeks and is characterized by non-specific symptoms. Almost all patients infected with *T. cruzi* enter an early chronic phase. While the majority of patients are asymptomatic in the acute phase, symptoms that appear are usually non-specific due to the parasitic invasion of the skin and parasitemia, such as dermatitis, edema, tachycardia, fever, malaise and headache. Of note, ~1 in every 5 individuals will progress to a symptomatic chronic phase characterized by cardiomyopathy, megaesophagus and megacolon. Some individuals can present the involvement of the nervous system during the acute phase or the reactivation during chronic disease (4,14,40,41).

### Central nervous system manifestations

CNS manifestations of neurochagas encompass a broad spectrum of neurological complications resulting from *T. cruzi* invasion and subsequent inflammatory responses. Although these manifestations can vary in severity and presentation, they collectively contribute to significant morbidity and mortality in affected individuals. The mortality related for CNS Chagas disease is high. It depends on the disease phase, in which the mortality is almost 10% in the acute phase and >80% during reactivation (4,6). Shelton and Gonzalez (6) reviewed the literature regarding the neurological symptoms of individuals with CNS Chagas disease. They found, in order of frequency, motor deficit, seizures, altered mental status, focal neurologic deficits, meningismus, dysarthria, aphasia, ataxia, disorientation, facio-brachio-crural paresis, intracranial hypertension signs, hemianopsia, Babinski sign and hyperreflexia (6).

One of the most extensively studied neurological manifestations associated with Chagas disease is stroke. Studies examining the risk factors for stroke in patients with Chagas disease are summarized in Table IV (2,3,5,7-9,18,22,41-46). Some individuals with neurochagas may develop stroke-like syndromes characterized by focal neurological deficits, such as hemiparesis, dysphasia, or visual disturbances. These deficits may result from ischemic or hemorrhagic strokes secondary to vasculopathy, thromboembolic events, or inflammatory vasculitis affecting cerebral blood vessels. Classically, stroke etiology in patients with Chagas disease is considered to be cardioembolic due to cardiac structural diseases, such as apical aneurysm and mural thrombus. Other etiologies have been observed in Chagas disease, and it is deemed that pro-inflammatory and prothrombotic disease states are the main factors associated with these etiologies. Factors associated with stroke recurrence and stroke mortality in Chagas disease are summarized in Table V (2,41,45).

Meningoencephalitis may present with symptoms, such as headache, fever, altered mental status, seizures and focal neurological deficits. Meningoencephalitis can result from direct parasite invasion, immune-mediated responses, or secondary infections. Notably, seizures have been reported with Chagas disease. They can manifest as focal and generalized epileptic events. Seizures may result from neuronal hyperexcitability, cortical scarring, or inflammatory processes affecting brain regions involved in seizure generation and propagation (4,14,40,41). Notably, meningoencephalitis is considered the most common symptom of acute neurological involvement in Chagas disease, and also occurs during reactivation, prompting urgent treatment; otherwise, it is almost fatal (46,47).

Cognitive impairment and dementia, manifesting as deficits in memory, attention, executive function and visuospatial abilities, are frequently observed in individuals with chronic infection with Chagas disease. These cognitive deficits may result from neuronal dysfunction, white matter abnormalities and neuroinflammatory processes affecting cortical and subcortical brain regions (48). These individuals usually have other clinical manifestations of Chagas, such as megacolon and congestive heart failure, at the time of their diagnosis of cognitive impairment associated with Chagas disease. Furthermore, various movement disorders associated with Chagas disease have already been reported, including Parkinsonism, chorea, dystonia and tremor. These movement disorders may result from basal ganglia dysfunction, dopaminergic depletion, or the disruption of corticostriatal circuits secondary to neuronal injury and neuroinflammation (23,49).

Psychiatric symptoms, such as depression, anxiety, psychosis and personality changes, are common in neurochagas and can significantly impair the social functioning and quality of life of affected individuals. These psychiatric manifestations may result from the direct effects of *T. cruzi* on brain neurotransmitter systems, neuroinflammatory responses, or psychosocial factors related to chronic illness and disability (50,51).

### Peripheral nervous system manifestations

Peripheral neuropathy is a common complication of chronic-stage neurochagas, characterized by damage to peripheral nerves, resulting in sensory, motor, or mixed neuropathies. Peripheral neuropathy in neurochagas often manifests asymmetrically and may involve both upper and lower extremities. Neurochagas can also lead to autonomic nervous system dysfunction, resulting in various symptoms affecting cardiovascular, gastrointestinal, genitourinary and sudomotor function (50,52,54-56).

Rare cases of cranial neuropathies associated with Chagas disease have already been reported. The most frequently affected cranial nerves include the trigeminal and oculomotor nerves. The involvement of other cranial nerves, such as the facial or vestibulocochlear nerve, may also occur (50,54-56).

Neurochagas can affect the neuromuscular junction, leading to myasthenia gravis-like symptoms, such as weakness, fatigue and fluctuating muscle strength. The dysfunction of the neuromuscular junction may result from autoimmune mechanisms, antibody-mediated injury, or direct effects of *T. cruzi* on neuromuscular transmission. Neurochagas may cause sensory abnormalities, including hypoesthesia, hyperesthesia, or neuropathic pain, affecting dermatomes or peripheral nerve distributions (23,49,55).

## 6. Diagnosis of neurochagas

Diagnosing neurochagas requires a comprehensive approach integrating clinical evaluation, laboratory investigations, neuroimaging studies and cerebrospinal fluid (CSF) analysis. Due to the diverse range of neurological complications associated with neurochagas and the lack of specific diagnostic tests, a multidimensional assessment is essential to establish an accurate diagnosis and guide appropriate management strategies (56,57).

As is classic in clinical settings, a thorough clinical history, and history of travel or zoonotic diseases, as well as a physical examination are essential for identifying neurological symptoms and signs suggestive of neurochagas. Clinicians should also assess risk factors, such as residence in endemic areas, exposure to triatomine bugs, and prior diagnosis of Chagas disease (6,23,54,57).

Serological tests are the primary method for diagnosing Chagas disease and detecting *T. cruzi* infection. Enzyme-linked immunosorbent assay, indirect immunofluorescence assay and western blot analyses are commonly used to detect antibodies against *T. cruzi* antigens in serum or plasma samples (57). Although positive serology indicates exposure to *T. cruzi*, it does not differentiate between acute and chronic infection. PCR assays can detect *T. cruzi* DNA in blood, CSF, or tissue samples, providing direct evidence of parasite presence and active infection. PCR may be particularly useful for diagnosing acute or reactivated Chagas disease and detecting parasite DNA in the CSF of individuals with neurochagas (58,59).

Neuroimaging studies may be performed to evaluate structural abnormalities in the brain and spinal cord associated with neurochagas. Imaging findings may include meningeal enhancement, parenchymal lesions, white matter abnormalities, infarcts and atrophy (59-61). A CSF examination may reveal pleocytosis, elevated protein levels, and the presence of oligoclonal bands or *T. cruzi* antibodies, supporting the diagnosis of neurochagas. Additional tests, such as neurophysiological studies, neuropsychological testing and autonomic function tests, may be performed to assess neurological function. These tests can provide valuable information about the extent and severity of neurological impairment in individuals with neurochagas (4,14,57,58).

## 7. Treatment strategies

The treatment strategies for neurochagas aim to alleviate symptoms, reduce parasite burden, mitigate neuroinflammation and improve overall clinical outcomes. However, treatment options for neurochagas remain limited, and management is often focused on symptomatic relief and supportive care (4).

### Antiparasitic therapy

Benznidazole is a first-line antiparasitic agent commonly used in the treatment of Chagas disease. It is a nitroimidazole derivative that disrupts the replication and metabolism of *T. cruzi* parasites. Benznidazole has exhibited efficacy in reducing parasite burden and preventing disease progression in individuals with acute and early chronic Chagas disease. However, its effectiveness in treating neurochagas, particularly in individuals with advanced neurological complications, remains uncertain (60,61). While benznidazole has been shown to clear *T. cruzi* in PCR-positive patients with non-neurological chronic Chagas disease, this clearance does not necessarily translate into clinical benefit for the patients (4,57,61).

Nifurtimox is another antiparasitic agent used to treat Chagas disease. As with benznidazole, nifurtimox is a nitroimidazole compound that exerts trypanocidal effects by generating toxic free radicals within *T. cruzi* parasites. While nifurtimox has demonstrated efficacy in treating acute and early chronic Chagas disease, limited data regarding its effectiveness in managing neurochagas are available (4,57,61).

The detailed description of antiparasitic choices is presented in Table VI (64-76). While antiparasitic drugs may exhibit efficacy in Chagas disease, their efficacy in cases with neurochagas is limited by the limited available clinical data on such efficacy in human patients, as well as the extent of their penetration to the CNS, apart from their known adverse events.

### Symptomatic management

Analgesic medications, such as non-steroidal anti-inflammatory drugs, opioids and neuropathic pain agents, may be prescribed to alleviate neuropathic pain and discomfort associated with neurochagas. Antiseizure medications may be used to control seizures and manage epilepsy in individuals with neurochagas. Antidepressants and anxiolytics may be prescribed to manage depression, anxiety and other psychiatric symptoms associated with neurochagas (4,53,57).

### Immunomodulatory therapy

Corticosteroid medications may suppress neuroinflammatory responses and alleviate symptoms in individuals with neurochagas-associated meningoencephalitis, vasculitis, or other autoimmune-mediated complications. However, corticosteroids should be carefully monitored due to the risk of exacerbating immunosuppression and promoting parasite dissemination. Furthermore, immunomodulatory agents may be considered in selected cases of neurochagas with autoimmune or inflammatory etiologies. These agents aim to modulate immune responses and attenuate neuroinflammation, but should be used judiciously and with caution (77-79).

## 8. Limitations of the current evidence and review

Although the first to comprehensively review the topic of neurochagas, the present review does have some limitations. The present study applied a narrative synthesis without restrict systematic search or selection process, which could be a source of selection bias. The lack of clinical evidence specifically in the part of clinical management is a major drawback in this topic. Furthermore, the available medications are limited by the poor or unknown penetration of the nervous system and, hence, the measurable efficacy in clinical settings. Moreover, the under- or misdiagnosis complicates the early diagnosis and treatment, and hence hardens the clinical benefit of treatment.

## 9. Future perspectives and research directions

Future perspectives and research directions in the field of neurochagas encompass a wide range of areas aimed at advancing the understanding of disease pathogenesis, improving diagnostic methods, developing novel treatment strategies, and enhancing patient care. Further elucidating the molecular and cellular mechanisms underlying neurochagas, including parasite-host interactions, neuroinflammatory processes, and neuronal damage pathways, is essential for identifying novel therapeutic targets and developing targeted interventions to mitigate disease progression (4,76). While the involvement of the CNS in zoonotic diseases is documented, the detailed descriptions of cases and diseases are not that clear or common. A detailed description of neuro-zoonotic diseases is encouraged.

Identifying reliable biomarkers for neurochagas, including serological, neuroimaging and CSF markers, is crucial for improving early diagnosis, monitoring disease progression and predicting treatment response. Biomarker discovery efforts should focus on identifying specific markers of neuronal injury, neuroinflammation and treatment efficacy in individuals with neurochagas. Advancing neuroimaging techniques can provide valuable insight into structural and functional abnormalities associated with neurochagas. Longitudinal imaging studies are required to characterize disease progression, identify imaging biomarkers and assess treatment effects in affected individuals. While specific details on sequelae of cases with neurochagas are lacking, specific details of uncommon cases are encouraged, with relevant information on history, examination, diagnosis and management.

Investigating the role of immunomodulatory agents, including cytokine inhibitors, monoclonal antibodies and immune checkpoint inhibitors, in the management of neurochagas-associated neuroinflammation and autoimmune complications represents a promising avenue for future research. Preclinical and clinical studies are warranted to evaluate the safety, efficacy and long-term outcomes of immunomodulatory therapies in individuals with neurochagas. Furthermore, developing novel antiparasitic agents with improved efficacy, safety and tolerability profiles for treating neurochagas remains a critical research priority. Drug discovery efforts should focus on identifying novel drug targets, repurposing existing drugs, and optimizing drug delivery strategies to enhance parasite clearance and prevent disease progression (75,76).

Implementing precision medicine approaches, including genomics, proteomics and metabolomics, can help identify individualized treatment strategies and predict treatment responses in individuals with neurochagas. Integrating molecular profiling data with clinical parameters can facilitate personalized treatment decisions and optimize patient outcomes.

Strengthening global health initiatives and partnerships to raise awareness, improve access to healthcare services, and enhance surveillance and control efforts for Chagas disease is critical for reducing the burden of neurochagas worldwide. The World Health Organization (WHO) has supported successful initiatives, for example, starting from Latin America, for screening of Chagas disease and early access to treatment (80,81). The early screening of susceptible personnel (e.g., transplant patients) and initiatives in non-endemic areas with high rates of immigrants are urged to avoid the delayed recognition and clinical deterioration. Collaborative efforts between endemic countries, international organizations and research institutions are required to address the socioeconomic, cultural and structural barriers to effective Chagas disease control.

## 10. Conclusion

In conclusion, neurochagas represent a critical public health challenge, with devastating neurological consequences for affected individuals worldwide. Despite advances in the understanding of the disease pathogenesis and management strategies, considerable gaps remain to be addressed in the current knowledge of neurochagas. The complex interplay between *T. cruzi* infection, immune responses and neuroinflammatory processes underscores the need for multidisciplinary research efforts to elucidate disease mechanisms, identify biomarkers and develop effective treatments. Future research is required to prioritize identifying reliable biomarkers for early diagnosis, disease monitoring and treatment response assessment in individuals with neurochagas. Moreover, global health initiatives should be strengthened to raise awareness, improve access to healthcare services, and enhance surveillance and control efforts for Chagas disease.

## Figures and Tables

**Table I:** Prevalence and incidence of Chagas disease.

Region	Prevalence	Incidence	Considerations
Latin America	High (6-7 million cases)	Varied	Chagas disease is endemic in Latin America, with an estimated 6-7 million cases, but the incidence varies.
North America	Low (few thousand cases)	Low	Chagas disease is increasingly recognized in North America, but prevalence and incidence remain low. In the USA, Chagas disease risk is higher in southern states (where there are more kissing bugs) than in northern states.
Europe	Low (few hundred cases)	Low	Chagas disease is rare in Europe, primarily affecting migrants from endemic regions.
Asia, Africa, and global	Limited data	Limited data	Limited data on the prevalence and incidence of Chagas disease in Asia, primarily among travelers or migrants.

The information presented in the table was obtained from previous studies (4-6,8,14,15).

**Table II:** Rodent models exhibiting CNS abnormalities following *Trypanosoma cruzi* infection.

Rodent model	*Trypanosoma cruzi* strain	Infection route	CNS infected region	Author(s), year of publication	(Refs.)
C3H/HeJ mice	Tulahuen	Subcutaneous	Brain	Buckner *et al*, 1999	(20)
C3H/HeJ mice	Colombian	Intraperitoneal	Meningoencephalitis, choroid plexus, and hippocampus	Silva *et al*, 1999	(32)
Swiss mice	Colombian	Intraperitoneal	Meninges, cerebral cortex	Baldissera *et al*, 2017	(82)
Holtzman rats	Y, CL, PNM	Intraperitoneal	Gray and white matter in cerebral and cerebellar cortices, astrocytes	Da Mata *et al*, 2000	(17)
C57BL/6 mice	Colombian	Intraperitoneal	Meningoencephalitis, leptomeninges, parenchyma, choroid plexus, cerebellum	Roffê *et al*, 2003	(33)
BALB/c mice	Dm28c	Oral	Olfactory bulb, pituitary gland	Silva-Dos-Santos *et al*, 2017	(10)
C3H/HeJ mice C57BL/6 mice	Colombian	Intraperitoneal	Parenchyma, hippocampus, cerebellum, astrocytes, and microglia	Vilar-Pereira *et al*, 2012	(34)
Wistar rats	Y	Intraperitoneal	Hypothalamus	Jardim, 1971	(35)
C57BL/6 mice	Tulahuen	Intranasal	Basal ganglia, cortex cerebellum	Caradonna and Pereiraperrin, 2009	(19)
BALB/c mice	Colombian	Intraperitoneal	Pituitary gland	Corrêa-de-Santana *et al*, 2005	(36)

**Table III:** Suggested molecular interactions in conditions such as neurochagas and host molecules in the CNS.

Interacting host protein	Interacting protein location in the human CNS	*T. cruzi*-derived protein	Author(s), year of publication	(Refs.)
Kininogen	CNS	Cruzipain	Scharfstein *et al*, 2000	(25)
TGF-β receptor	Glia, neuron	TGF-β-like molecule	Useche *et al*, 2022	(23)
Galectin 3	Microglia	β-galactose glycoconjugates	Quenum Zangbede *et al*, 2018	(37)
TrkA	Neurons, dendritic cells, astrocytes, and microglia	Trans-sialidase	Chuenkova and Pereira, 2001	(38)
TrkC	Neurons, dendritic cells	Trans-sialidase	Weinkauf and Pereiraperrin, 2009	(39)
Cytokeratin 18 - Laminin	Blood-brain-barrier	Tc85	De-Souza *et al*, 2010 and Useche *et al*, 2022	(23,83)
Heparan sulfateproteoglycan	CNS	Penetrin	O'Callaghan *et al*, 2018 and Useche *et al*, 2022	(21,23)
Heparin G proteins, fibronectin, and human type I and IV collagen	Human brain microvascular endothelial cells	Tc80 (oligopeptidase B)	Silva *et al*, 1999 and Useche *et al*, 2022	(23,32)

CNS, central nervous system; *T. cruzi*, *Trypanosoma cruzi*; Tc80, *T. cruzi* 80-kDa proteinase; Tc85, *T. cruzi* 85-kDa proteinase; TGF-β, transforming growth factor-β; TrkA, tropomyosin receptor kinase A; TrkC, tropomyosin receptor kinase C.

**Table IV:** Studies on stroke associated with Chagas disease.

Author(s), year of publication	Sample size	No. of individual with stroke	Comments	(Refs.)
Bestetti, 2000	79	1	Low prevalence of stroke related to Chagas disease.	(5)
Aras *et al*, 2003	524	92	All the individuals were autopsied. Cerebral infarction was associated with death in 52% of the cases.	(9)
Oliveira-Filho *et al*, 2005	305	32	Systolic dysfunctions, presence of cardiac arrhythmias, cardioversion, and diabetes are predictors of stroke.	(42)
Carod-Artal *et al*, 2005	478	94	Apical aneurysms, heart failure, arrhythmia, female, and hypertension are predictors of stroke.	(2)
Paixão *et al*, 2009	101	101	Previous stroke/transient ischemic attack history, atrial fibrillation, and CD-positive serology are associated with stroke.	(3)
Nunes *et al*, 2009	213	39	Left ventricular systolic dysfunction and left atrial volume enlargement are independent risk factors for stroke.	(40)
Jesus *et al*, 2011	144	9	Chagas disease and stroke history are risk factors for microembolism.	(18)
Dias Junior *et al*, 2014	52	26	Apical aneurysms and intracavitary thrombus.	(7)
Nunes *et al*, 2015	330	67	Apical aneurysm and left ventricular thrombus.	(43)
Guedes *et al*, 2016	65	35	Thromboembolic events, imbalanced. Expression of IL-10, FoxP3, and iNOS are associated with higher stroke and death risks.	(22)
Montanaro *et al*, 2018	279	279	Age at stroke, initial modified Rankin Scale, bladder dysfunction, diabetes, and alcoholism are associated with mortality after stroke.	(41)
Montanaro *et al*, 2021	499	499	Higher prevalence of vascular risk factors and lower median age in patients with cardioembolic etiology.	(44)
Cerqueira-Silva *et al*, 2022	271	16	Increased risk of stroke and death when compared to other etiologies of heart failure, independently of HF severity or cardiac structural diseases.	(8)

**Table V:** Factors associated with stroke recurrence and stroke mortality in Chagas disease.

Factors associated with stroke recurrence	Factors associated with stroke mortality	Factors for cardioembolic stroke in Chagas disease
Risk factors: Cardio-aortic embolic etiology; initial modified Rankin scale >3 at admission; female sex	Risk factors: Age at stroke; bladder dysfunction; diabetes mellitus; initial modified Rankin scale >3 at admission	Cardiomyopathy: Left atrium dilatation; progressive heart failure (left ventricular systolic or diastolic); segmental lesions (left ventricular posterior wall lesion and apical aneurysm)
Protective factor: Age at stroke; cognitive deficit after stroke		Arrhythmias: Right bundle branch block often associated with left anterior hemiblock; advanced atrioventricular block; atrial fibrillation; sustained ventricular tachycardia Mural thrombus

The information presented in the table was obtained from previous studies (2,5,8,41,45).

**Table VI:** Antiparasitic choices for managing neurochagas.

Antiparasitic drug	Mechanism of action	Administration	Efficacy	Adverse effects	Comments	(Refs.)
Benznidazole	Inhibition of trypanosomal enzymes	Oral	Variable; limited CNS penetration	Gastrointestinal disturbances, rash, neurotoxicity	First-line treatment for Chagas disease, including neurochagas. May require prolonged therapy	(4,57,61)
Nifurtimox	Formation of toxic metabolites	Oral	Variable; limited CNS penetration	Gastrointestinal disturbances, rash, neurotoxicity	Alternative option for Chagas disease treatment, less commonly used due to tolerability issues	(4,57,61)
Fexinidazole	Inhibition of trypanosomal enzymes	Oral	Limited clinical data	Gastrointestinal disturbances, hepatotoxicity, QT prolongation, neurotoxicity	Investigational drug for Chagas disease, undergoing clinical trials for safety and efficacy.	(62,63)
Posaconazole	Inhibition of trypanosomal sterol synthesis	Oral	Limited clinical data	Gastrointestinal disturbances, hepatotoxicity, QT prolongation, neurotoxicity	Effective in preclinical studies, but require further clinical investigation.	(66,68,69)
Ravuconazole	Inhibition of trypanosomal sterol synthesis	Oral	Limited clinical data	Gastrointestinal disturbances, hepatotoxicity, QT prolongation, neurotoxicity	Investigational drug undergoing preclinical evaluation for Chagas disease treatment.	(64,65,67,84)
Arsenical Rosaniline dye antimonials mercury chloride	Depends on the *Trypanosoma cruzi* strain	Oral	Limited clinical data	Unknown	No effects. Historical use.	(72,74)
Allopurinol	Interfering with RNA synthesis	Oral	Limited clinical data	Gastrointestinal symptoms, headache, dark urine, jaundice, and muscle weakness.	Ineffective during the acute phase.	(70,71,73,74)

CNS, central nervous system.

## Data Availability

Not applicable.
